# RNA N6-Methyladenosine Modifications and Its Roles in Alzheimer’s Disease

**DOI:** 10.3389/fncel.2022.820378

**Published:** 2022-03-24

**Authors:** Runjiao Zhang, Yizhou Zhang, Fangzhen Guo, Sha Li, Huixian Cui

**Affiliations:** ^1^Department of Anatomy, Hebei Medical University, Shijiazhuang, China; ^2^Neuroscience Research Center, Hebei Medical University, Shijiazhuang, China; ^3^Hebei Key Laboratory of Neurodegenerative Disease Mechanism, Shijiazhuang, China

**Keywords:** Alzheimer’s disease, demethylase, methyltransferase, methylation-binding protein, memory disorder, N6-methyladenosine

## Abstract

The importance of epitranscriptomics in regulating gene expression has received widespread attention. Recently, RNA methylation modifications, particularly N6-methyladenosine (m^6^A), have received marked attention. m^6^A, the most common and abundant type of eukaryotic methylation modification in RNAs, is a dynamic reversible modification that regulates nuclear splicing, stability, translation, and subcellular localization of RNAs. These processes are involved in the occurrence and development of many diseases. An increasing number of studies have focused on the role of m^6^A modification in Alzheimer’s disease, which is the most common neurodegenerative disease. This review focuses on the general features, mechanisms, and functions of m^6^A methylation modification and its role in Alzheimer’s disease.

## Introduction

**FIGURE 1:**
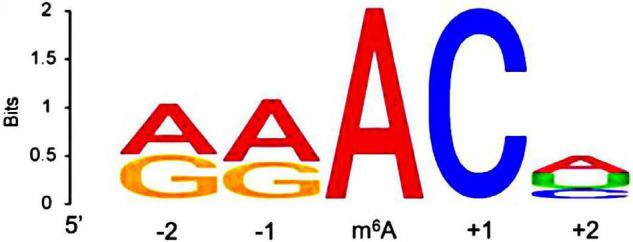
The canonical RRACH motif. R, adenine or guanine; H, cytosine, adenine, or uracil.

Epitranscriptomics, i.e., chemical modification used for RNA regulation, has recently emerged as a highly investigated subfield of neuroscience. In the early 1970s, RNA methylation modifications were discovered (Desrosiers et al., 1974). These include N6-methyladenosine (m^6^A), N1-methyladenosine (m^1^A), N6, 2-O-dimethyladenosine (m^6^Am), 5-methylcytosine (m^5^C), 5-hydroxymethylcytosine (5hmC), and 7-methylguanine (m^7^G) (Chen et al., 2016). Among them, m^6^A is the most common and abundant type of eukaryotic methylation modification in RNAs, including mRNAs, long non-coding RNAs (lncRNAs), circular RNAs (circRNAs), microRNAs (miRNAs), rRNAs, tRNAs, and small nuclear RNAs (snRNAs) (Yue et al., 2015; Du et al., 2018; Shi et al., 2019). The adenine in the RRACH sequence (R = adenine or guanine, and H = cytosine, adenine, or uracil) is usually the site of m^6^A modification (Figure 1; Deng et al., 2018). In mammals, m^6^A methylation modification is widely distributed in many tissues, particularly in the brain (Meyer and Jaffrey, 2014). The abundance of m^6^A and its emerging role as an important post-transcriptional regulator in the mammalian brain has gained wide attention in the field of neuroepigenetics (Chang et al., 2017).

Alzheimer’s disease (AD), the most frequently and commonly diagnosed dementia, is an increasing global health concern with a notable impact on human health (Guo et al., 2020). Despite significant advances in our understanding of AD pathogenesis and the definition of the disease since the first case reported by Alzheimer (1907), there are still no disease-modifying treatments (Pakavathkumar et al., 2017). Altered m^6^A-methylation has been considered associated with AD as many players in the m^6^A pathway have been implicated as critical factors in neuronal function (Shafik et al., 2021).

In this review, we will focus on the general features, functions, and adjustability of m^6^A methylation modifications and their role in AD.

## RNA N6-Methyladenosine Methylation Modification Protein

The m^6^A modification of RNA has been proven to be reversible, as it is bidirectionally regulated by m^6^A methyltransferase and demethylase, which work with RNA m^6^A methylation-binding protein to regulate the fate of RNAs (Knuckles and Bühler, 2018; Widagdo and Anggono, 2018). These proteins have served as valuable tools for investigating the cellular and physiological roles of m^6^A methylation modification in the brain.

### N6-Methyladenosine Methyltransferase

The m^6^A methyltransferases, also known as “Writers” in RNA m^6^A methylation modification, catalyze the transfer of a methyl group from S-adenosyl methionine (SAM) to adenine nucleotides of RNA substrates. Some “Writers,” including methyltransferases such as 3/14 (METTL3/14), Wilms’ tumor 1-associating protein (WTAP), KIAA1429, and RNA-binding motifs protein 15/15B (RBM15/15B), form the core components of m^6^A methyltransferase, which work together to catalyze methylation of RNA substrates (Liu et al., 2020). In addition, zinc finger CCCH-type containing 13 (ZC3H13) and E3 ubiquitin-protein ligase Hakai (HAKAI) are also components of this complex. Moreover, recent studies have found that methyltransferase-like 16 (METTL16) can catalyze the methylation of target RNAs alone, without relying on the above m^6^A methyltransferase complexes (Pendleton et al., 2017).

**FIGURE 2:**
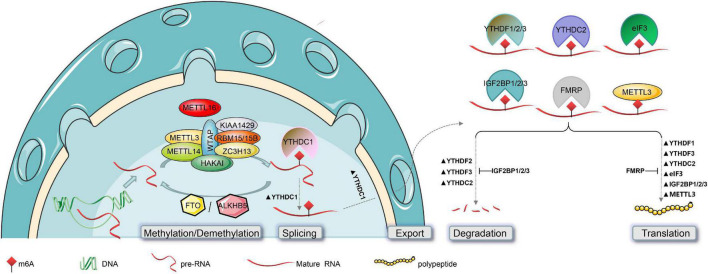
Post-transcriptional modification of RNA by m^6^A and its functions. The biogenesis of m^6^A in the mammalian nucleus is catalyzed by the methyltransferase complex (composed of METTL3, METTL14, WTAP, KIAA1429, RBM15/15B, ZC3H13, and HAKAI) or METTL16, and is reversed by m^6^A-demethylase (FTO or ALKBH5). The functional effects of m^6^A on RNAs can affect their nuclear splicing, nuclear export, stability, and translation efficiency. This is mediated by m^6^A “Readers,” such as YTHDC1 in the nucleus, and YTHDF1/2/3, YTHDC2, eIF3, IGF2BP1/2/3, and FMRP in the cytoplasm. Notably, in addition to catalyzing m^6^A methylation, METTL3 also directly promotes RNA translation.

**FIGURE 3:**
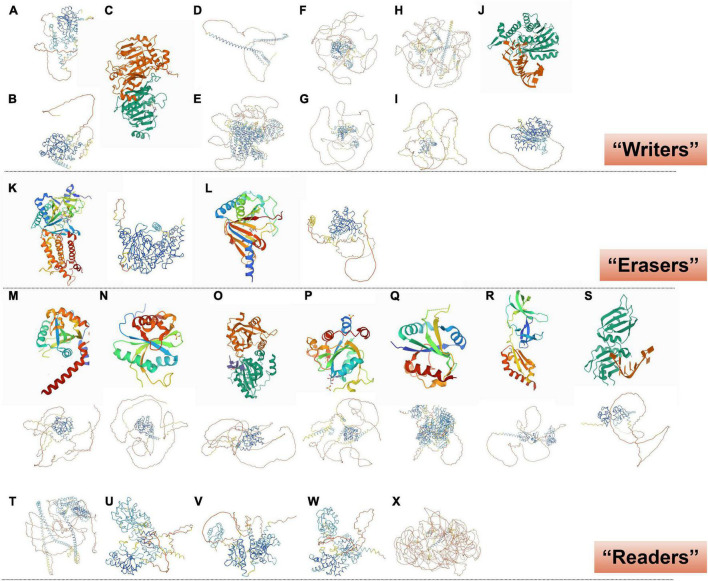
Crystal structure of RNA m^6^A methylation-modification enzymes. **(A)** METTL3 and **(B)** METTL14, predicted from “AlphaFold.” **(C)** SAM-bound METTL3-METTL14 complex (Wang et al., 2016). **(D)** WTAP, **(E)** KIAA1429, **(F)** RBM15, **(G)** RBM15B, **(H)** ZC3H13, and **(I)** HAKAI, predicted from “AlphaFold.” **(J)** Human METTL16 catalytic domain in complex with MAT2A 3’-UTR hairpin 6 (Upper; Doxtader et al., 2018); METTL16, predicted from “AlphaFold” (Lower). **(K)** FTO reveals the basis for its substrate specificity (Left; Han et al., 2010); FTO, predicted from “AlphaFold” (Right). **(L)** Human ALKBH5 in complex with citrate and acetate (Left; Feng et al., 2014); ALKBH5, predicted from “AlphaFold” (Right). **(M)** YTHDF1 YTH domain (Upper; Xu et al., 2015); YTHDF1, predicted from “AlphaFold” (Lower). **(N)** YTH-YTHDF2 in the free state (Upper; Li et al., 2014); YTHDF2, predicted from “AlphaFold” (Lower). **(O)** YTHDF3 YTH domain in complex with m^6^A RNA (Upper; Li et al., 2020); YTHDF3, predicted from “AlphaFold” (Lower). **(P)** YTHDC1 with m^6^A (Upper; Li Y. et al., 2021); YTHDC1, predicted from “AlphaFold” (Lower). **(Q)** Human YTHDC2 YTH domain (Upper; Ma et al., 2019); YTHDC2, predicted from “AlphaFold” (Lower). **(R)** N-terminal domain of FMRP (Upper; Myrick et al., 2015); FMRP, predicted from “AlphaFold” (Lower). **(S)** HNRNPA2B1 in complex with RNA (Upper; Wu et al., 2018); HNRNPA2B1, predicted from “AlphaFold” (Lower). **(T)** Eif3, **(U)** IGF2BP1, **(V)** IGF2BP2, **(W)** IGF2BP3, and **(X)** PRRC2A, predicted from “AlphaFold.”

METTL3 (Figures 2, 3A) is the best-known m^6^A methyltransferase. It is identified as a SAM-binding component in the complex and has its own catalytic ability. Unlike METTL3, METTL14 (Figures 2, 3B) does not bind to the SAM domain performing its own m^6^A methyltransferase catalytic ability but plays a key role in substrate identification. Biochemical and structural studies have revealed that METTL3 and METTL14 form a heterodimer (Figure 3C; Wang et al., 2016) that has a higher methylation activity than METTL3 alone (Liu et al., 2014; Wang et al., 2017). WTAP (Figures 2, 3D) is a regulatory subunit of the complex that interacts with METTL3 and METTL14 and localizes the METTL3-METTL14 complex to the nucleus (Ping et al., 2014; Schwartz et al., 2014). KIAA1429 (Figures 2, 3E) can mediate preferential mRNA methylation in 3’UTR and near stop codon and is also known as vir-like m^6^A methyltransferase-associated (VIRMA), whose N-terminus has the ability to recruit the METTL3/METTL14/WTAP complex (Yue et al., 2018). RBM15/15B (Figures 2, 3F,G) was three- to four-fold higher at the RRACH sequence site than at the non-methylation site. Knockdown of RBM15/15B decreases m^6^A levels in cellular mRNA (Knuckles et al., 2018). ZC3H13 (Figures 2, 3H) and HAKAI (Figures 2, 3I) are also components of the methyltransferase complex. ZC3H13 anchors the complex to the nucleus (Wen et al., 2018), and HAKAI regulates m^6^A levels in *Arabidopsis* (Růžička et al., 2017). Additionally, METTL16 (Figures 1, 3J; Doxtader et al., 2018) is also known as RNA m^6^A methyltransferase, an enzyme that maintains SAM homeostasis (Shima et al., 2017; Warda et al., 2017; Aoyama et al., 2020). METTL16 does not form complexes with other m^6^A methyltransferases, and has a distinct set of targets for m^6^A modification, including the 3′-untranslated region (UTR) of MAT2A mRNA and U6 snRNA.

N6-methyladenosine methyltransferases are mainly localized in the nucleus (Ping et al., 2014), which is consistent with the m^6^A-binding site found in nascent pre-mRNAs or pri-miRNAs (Ping et al., 2014; Ke et al., 2017; Slobodin et al., 2017). Interestingly, METTL3 catalyzes the methylation of mature mRNAs and has a non-methylating function in the cytoplasm (Lin et al., 2019; Liu et al., 2020). Lin et al. (2016) found that METTL3 was associated with ribosomes and promoted mRNA translation in the cytoplasm. Additionally, METTL16 functions independently of m^6^A methylation. In SAM-limiting conditions, METTL16 occupancy of a hairpin (hp1) in the MAT2A 3′-UTR induces splicing of the MAT2A-retained intron, which controls the production of SAM (Pendleton et al., 2017). Thus, while m^6^A methyltransferases generally affect RNA processing through m^6^A “Readers,” direct contributions of methyltransferases to RNA metabolism should not be overlooked (Pendleton et al., 2017; Doxtader et al., 2018).

### N6-Methyladenosine Demethylase

The m^6^A demethylases, also known as “Erasers” in RNA m^6^A methylation modification, catalyze the demethylation of RNA substrates modified by m6A. In eukaryotes, fat mass and obesity-associated protein (FTO) and AlkB homolog 5 (ALKBH5) were both found to be demethylases. These enzymes belong to the AlkB family of the dioxygenase superfamily and have a similar catalytic core, although they prefer different substrates and are expressed in different organs (Gerken et al., 2007; Zheng et al., 2013; Zou et al., 2016; Liu et al., 2020).

Fat mass and obesity (Figures 2, 3K; Han et al., 2010), also known as AlkB homolog 9 (ALKBH9), was the first discovered RNA m^6^A demethylase. FTO is mainly detected in the nucleus, similarly to the m^6^A methyltransferase (Jia et al., 2011). Its long stem-loop domain at the C-terminus enables substrate RNA demethylation (Jia et al., 2011; Bartosovic et al., 2017). However, Hess et al. (2013) only found 5,000 new m^6^A peaks in FTO-knockout mice, compared with 42,000 peaks in the control samples. These data indicated that FTO does not globally target all m6A-modified mRNAs, or which seems to imply that another m^6^A “Eraser,” ALKHB5, compensates for the lack of FTO. ALKBH5 (Figures 2, 3L) is similar to FTO and is also a Fe^2+^ and α-Ketoglutaric acid-dependent non-heme oxygenase (Zheng et al., 2013; Feng et al., 2014).

### RNA N6-Methyladenosine Methylation-Binding Protein

The m^6^A binding proteins, also known as “Readers,” in RNA m^6^A methylation modification, specifically bind to the m^6^A methylation region, weakening the homologous binding to RNA reading proteins, and altering the secondary structure of RNA to alter protein–RNA interaction (Li et al., 2019). This function is widely implicated in the stability, translation, alternative splicing, and subcellular targeting of specific RNAs by recruiting or repelling some RNA-binding proteins (RBPs) or by altering the secondary structure of targeted RNAs (Adhikari et al., 2016; Wu et al., 2017; Widagdo and Anggono, 2018). Many “Readers,” including YTH domain-containing RNA-binding protein (YTP), fragile X mental retardation protein (FMRP), heterogeneous nuclear ribonucleoprotein (HNRNP), eukaryotic initiation factor 3 (eIF3), insulin-like growth factor 2 mRNA-binding protein (IGF2BP), and proline-rich coiled-coil 2A (PRRC2A), have already been identified.

YTH domain-containing RNA-binding protein includes YTH domain-containing family protein 1/2/3 (YTHDF1/2/3) (Figures 2, 3M–O; Li et al., 2014, 2020; Xu et al., 2015) and YTH domain-containing protein 1/2 (YTHDC1/2) (Figures 2, 3P,Q; Ma et al., 2019; Li Y. et al., 2021). Their YTH domains are capable of combining with the m^6^A RRACH sites to mediate RNA-specific binding, while their proline/glutamine/asparagine-enriched (P/Q/N-rich) domains regulate the subcellular localization of target RNA (Liao et al., 2018; Patil et al., 2018). YTHDF1/2/3 and YTHDC2 play specific roles in the cytoplasm, and YTHDC1 plays a role in the nucleus. It is generally considered that YTHDF1 enhances mRNA translation by promoting ribosome occupancy and interacting with initiation factors, YTHDF2 promotes mRNA degradation by localizing m^6^A-modified RNA to mRNA decay sites, and YTHDF3 enhances translation along with YTHDF1 and promotes degradation along with YTHDF2 in the cytoplasm (Wang et al., 2014; Li A. et al., 2017; Shi et al., 2017, 2018). However, Zaccara and Jaffrey (2020) showed that YTHDF1/2/3 co-regulated mRNA degradation rather than promoting mRNA translation in HeLa cells. Similar to YTHDF3, YTHDC2 in the cytoplasm accelerates degradation of the modified mRNA and enhances the translation of the corresponding protein by recognizing m^6^A (Hsu et al., 2017). Additionally, YTHDC1 regulates m^6^A-dependent mRNA splicing by recruiting splicing factors and mediates the nuclear export of m^6^A methylated mRNAs by interacting with the nuclear export adaptor protein SRSF3 (Xu et al., 2014; Xiao et al., 2016; Roundtree et al., 2017). Briefly, YTHDF1/2/3 and YTHDC2 promote the metabolism of m^6^A-modified mRNAs, but YTHDC1 regulates their splicing (Figure 2; Wang et al., 2014; Xiao et al., 2016; Hsu et al., 2017; Li A. et al., 2017; Shi et al., 2017, 2018; Zaccara and Jaffrey, 2020).

Fragile X mental retardation protein, an RBP (Figures 2, 3R; Myrick et al., 2015), negatively regulates the translation of mRNAs by interacting with m^6^A sites and then recruits RNA-induced silencing complexes and some miRNAs to arrest ribosomal elongation (Darnell et al., 2011; Suhl et al., 2014; Richter et al., 2015; Arguello et al., 2017; Chang et al., 2017).

Heterogeneous nuclear ribonucleoprotein is a group of RBPs that includes nearly 30 proteins, named A1 to U, which can interact with each other to form a complex. The most studied heterogeneous nuclear ribonucleoprotein A2/B1 (HNRNPA2B1) (Figure 3S; Wu et al., 2018) binds directly to a set of nuclear transcripts with m^6^A marks, and activates downstream variable shear events of partial genes (Alarcón et al., 2015; Geissler et al., 2016).

The eIF3 protein (Figures 2, 3T) facilitates the translation of mRNA by binding to the m^6^A sites of mRNA 5′-UTRs directly. In addition, IGF2BP, including IGF2BP1/2/3 (Figures 2, 3U–W), promotes mRNA stability and translation by recognizing the GG (m^6^A) C sequence (Huang et al., 2018). PRRC2A (Figure 3X) stabilizes mRNA expression by binding to the consensus GGACU motif in the coding sequence (CDS) region of mRNA in an m^6^A-dependent manner (Wu et al., 2019).

## Adjustability of RNA N6-Methyladenosine Methylation

The dynamic nature of chemical modifications is an essential feature of functionality in the nervous system (Widagdo and Anggono, 2018). RNA m^6^A methylation, as the most abundant internal RNA modification, contributes markedly to this. Not surprisingly, RNA m^6^A methylation is precisely regulated.

### Activity-Dependent Regulation of N6-Methyladenosine

Previous studies have demonstrated that cellular m^6^A levels are dynamically regulated in response to hypoxia, heat shock, and ultraviolet irradiation in cells (Dominissini et al., 2012; Meyer et al., 2015; Zhou et al., 2015; Zhang et al., 2016; Xiang et al., 2017; Lu et al., 2019). In the mammalian central nervous system, stimulus-dependent regulation of m^6^A has recently been shown to occur in response to behavioral training, cell microenvironment changes, and nerve injury (Widagdo et al., 2016; Engel et al., 2018; Shi et al., 2018; Weng et al., 2018; Zhang et al., 2018).

KCl is used to activate neurons by increasing membrane potentials in cells and opening voltage-gated calcium ion channels in cell membranes (Rosen et al., 1994; Lakk et al., 2017). m^6^A methylation of RNAs is upregulated following administration of KCl to primary neuronal cultures (Widagdo et al., 2016). Behavioral training is another way to stimulate neurons. Widagdo et al. (2016) discovered that the percentage of m^6^A-occupancies RNAs increased significantly in the medial prefrontal cortex after cued fear conditioning. A similar increase in the levels of m^6^A methylation was also observed in the dorsal hippocampus following contextual fear conditioning (Walters et al., 2017). Interestingly, activity-dependent m^6^A RNA modification has been found to occur in many immediate early genes and synaptic plasticity-related transcripts (Widagdo et al., 2016; Shi et al., 2018; Zhang et al., 2018). However, acute restraint-stress reduced global m^6^A levels in the mouse prefrontal cortex (Engel et al., 2018), which requires further investigation.

### Tissue, Cellular/Subcellular, and Site-Specific Regulation of N6-Methyladenosine

More intriguingly, dynamic adjustability of m^6^A has been demonstrated in different brain regions, such as the prefrontal cortex, hippocampus, and amygdala (Widagdo et al., 2016; Walters et al., 2017; Engel et al., 2018). Shafik et al. (2021) revealed that this tissue specificity was most pronounced in the hypothalamus.

As different brain areas include different types of cells, it is reasonable to speculate that RNA m^6^A methylation may have cellular and subcellular specificity. Although the transcriptomic profiles of m^6^A in neuronal subpopulations have yet to be established, bioinformatics analysis showed RNA m^6^A methylation-enrichment in genes specific to neuronal subtypes (Chang et al., 2017), which implies a particular bias for m^6^A toward RNAs in neurons rather than in glial cells. Furthermore, the m^6^A-modified transcripts were widespread in a position distal from the neuronal cell body, indicating an interesting mode of m^6^A regulation outside of the nucleus. Indeed, some immunocytochemical assays and biochemical subcellular fractionation tests have revealed that some m^6^A “Writers” (METTL3, METTL14, METTL16), “Erasers” (FTO, ALKBH5), and “Readers” (YTHDF1/2/3) are present in the extra-somatic regions of neurons (Gershoni-Emek et al., 2016; Merkurjev et al., 2018; Yu et al., 2018; Nance et al., 2020). This localization of m^6^A “Writers” and “Erasers” in the extra-somatic regions may expedite the dynamic regulation efficiency of neurons (Widagdo and Anggono, 2018) in response to changes in the extracellular milieu, although most of the work may still be done in the soma. The localization of m^6^A in axons and its role in axonal growth has also been studied (Yu et al., 2018). An axonal elongation factor, Gap-43, was found to be an mRNA target of m^6^A. The local translation of Gap-43 was negatively modulated by m^6^A methylation and could be regulated by FTO in axons (Yu et al., 2018). In addition, the function of m^6^A has been observed in the synapses. In the pre-and postsynaptic compartments of neuron, most of the m^6^A target genes fell into the Gene Ontology functional terms “cell junction” and “synapse,” as well as surface receptor pathways, all of which maintain the functionality and integrity of synapses (Merkurjev et al., 2018).

**FIGURE 4:**
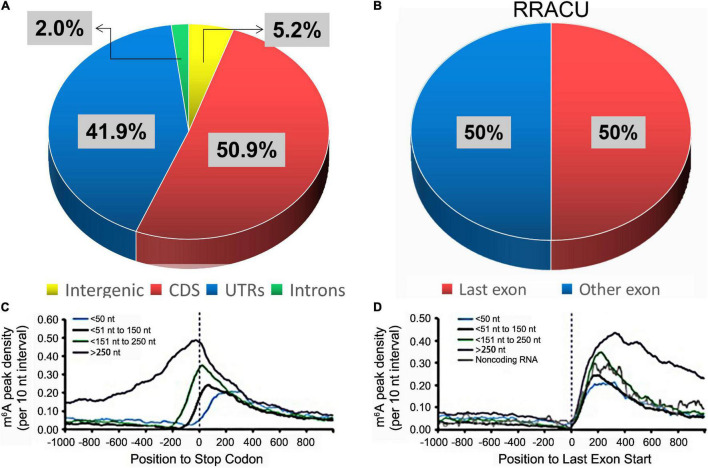
The distributions of m^6^A peaks. **(A)** The percentage of m^6^A peaks within distinct RNA sequence types. **(B)** The relative proportions of RRACU motifs in the last exon and other exons, with 100% representing all RRACU sequences in mRNA (Ke et al., 2015). **(C,D)** In the last exon, m^6^A density increased sharply (Ke et al., 2015). mRNAs were grouped according to their stop codon locations relative to the start of the last exon **(D)**.

Furthermore, the sites of m^6^A modification were found to be non-randomly distributed within genes (94.8%), where the proportions of CDS, UTRs, and introns were 50.9%, 41.9%, and 2.0%, respectively (Figure 4A; Meyer et al., 2012; Deng et al., 2018). Although the frequency of the RRACU sequence in last exons was the same as that in other exons (Figure 4B), m^6^A was found to be enriched within the last exons of a gene (Figures 4C,D; Ke et al., 2015).

### Developmental Stage-Specific Regulation of N6-Methyladenosine

Studies of m^6^A modifications have highlighted the need to maintain transcriptomic dynamics during neurodevelopment. The expression pattern of m^6^A indeed differs across various developmental phases (Meyer et al., 2012; Yoon et al., 2017). Shafik et al. (2021) also found that m6A exerted a critical function in both early and late brain development in a spatio-temporal fashion.

Prenatally, m^6^A modification is markedly increased throughout brain development (Meyer et al., 2012). Changing the m^6^A methylation modification by interfering with the expression levels of m^6^A “Writers” or “Erasers” leads to defects in brain development. The loss of m^6^A in embryonic neuronal progenitor cells by conditionally deleting METTL14 in embryonic mouse braiTablens resulted in delayed differentiation and prolonged cell cycle progression, extending cortical neurogenesis into the postnatal stages (Yoon et al., 2017). An abnormal increase of m^6^A by knocking out FTO caused cerebellar shrinkage and impaired spatial learning and memory (Li L. et al., 2017), which might be due to the increase in m^6^A promoting the decomposition of mRNAs encoding proteins with known functions in neuronal differentiation, stem cells, and the cell cycle (Yoon et al., 2017).

Postnatally, the function of m^6^A in neurogenesis was first uncovered in FTO-knockout mice (Li L. et al., 2017). Loss of FTO in FTO-knockout mice resulted in decreased proliferation and differentiation and reduced numbers of adult neural stem cells. In addition to the aforementioned central nervous system, loss of FTO in FTO-knockout mice also led to a shorter axonal length in mouse dorsal root ganglia neurons (Widagdo and Anggono, 2018). Taken together, these results suggest that balanced m^6^A modification plays a significant role in the establishment and development of the mammalian central and peripheral nervous systems.

### MicroRNA Regulation of N6-Methyladenosine-Related Enzymes

MicroRNAs, which are approximately 22-nucleotide single-strand sequences, are a group of important post-transcriptional regulators in eukaryotes, which affect RNA m^6^A methylation by targeting m^6^A “Writers,” “Erasers,” and “Readers” in terms of both their functions and expression. It has been reported that manipulation of some miRNA sequences or their expression altered m^6^A methylation modification levels by regulating the binding of METTL3 to some mRNAs containing miRNA-binding sites (Chen et al., 2015). In addition, miR-33a, which targets the METTL3 3′-UTR, suppressed cell proliferation (Liu et al., 2020). miRNA-421-3p targeting the “Reader” YTHDF1 inhibited p65 mRNA translation to prevent inflammatory responses in cerebral ischemia/reperfusion injury (Zheng et al., 2020). miR-145 restrained the expression of YTHDF2 by targeting YTHDF2 mRNA, thus inhibiting cell proliferation. miR-744-5p targeting the “Reader” hnRNPC promoted ovarian cancer cell death (Chen et al., 2020).

## RNA N6-Methyladenosine Methylation in Alzheimer’s Disease

**FIGURE 5:**
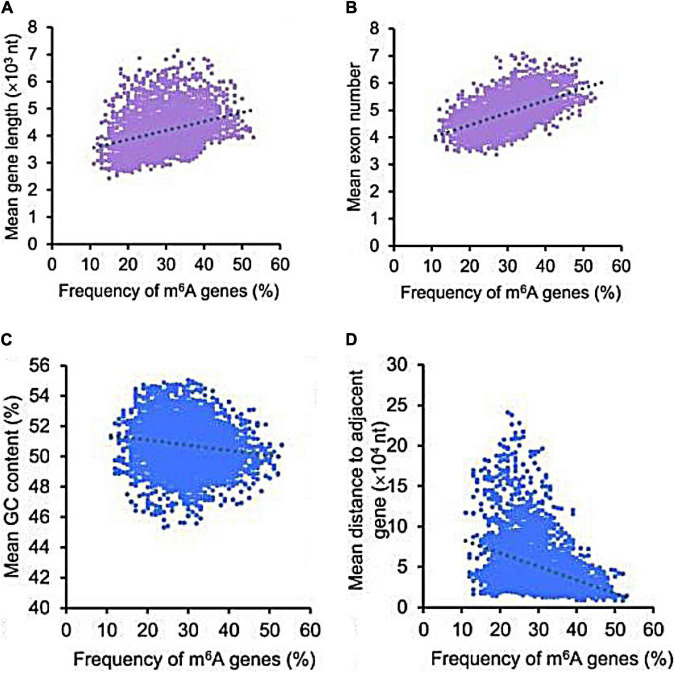
The correlation of m^6^A genes with multiple gene features. The frequency percentage of m^6^A genes was positively correlated with the length **(A)** and number of exons **(B)**, but was negatively correlated with GC content **(C)** and gene distance to the adjacent gene **(D)** (Miao et al., 2020).

Miao et al. (2020) observed that the frequency percentage of m^6^A in genes was positively correlated with the length and number of exons (Figures 5A,B) but negatively correlated with GC content and gene distance to the adjacent gene (Figures 5C,D), which implies that RNA m^6^A methylation is not random and disordered. Several recent reports have started to uncover the functional significance of m^6^A regulation in *de novo* RNA transcripts, including nuclear splicing, stability, translation, and subcellular localization, suggesting that m^6^A serves as a regulator to fine-tune many diseases precisely over time (Li et al., 2019). Indeed, m^6^A has been identified as a conserved epitranscriptomic modification in many neurodegenerative diseases, such as AD (Hess et al., 2013; Engel and Chen, 2018; Shafik et al., 2021).

Just as m^6^A methylation, AD has tissue, cellular/subcellular, and site-specificity, associated with environment and neurodevelopment and regulated by miRNAs. The progressive degeneration of hippocampal neurons is the main feature of AD. Mutations in the APP, PS1, and PS2 genes are considered the main causes of familial AD (Popugaeva et al., 2018). Environmental stimulation, such as infections, trauma, and radiofrequency radiation (Dasdag et al., 2020), is thought to induce sporadic AD. Neurodevelopment-related signaling pathways, such as Notch/Wnt/Reelin intracellular signaling pathways, may represent a novel approach to the regulation of neurodegenerative processes in AD (Grilli et al., 2003). In addition, the above-mentioned miRNAs that regulate m^6^A are also considered to be involved in the pathogenesis of AD. Jaouen and Gascon (2016) described miR-33 function modulating ATP-binding cassette transporter A1 (ABCA1) and interfering with Aβ plaque formation through cholesterol metabolism regulation. Peña-Bautista et al. (2021) found hsa-miR-421 showed a positive correlation with some detected lipids (FA (16:0), FA (20:2), FA (18:2), FA (20:4), FA (20:3), FA (18:0), FA (14:0)) in AD plasma samples. Docosahexaenoic acids (DHA) are known to be beneficial in AD. miR-33a and miR-145 are regulated by DHA, and this regulation becomes disrupted in AD (Chiang, 2021).

RNA m^6^A methylation has been considered to be an important epigenetic marker associated with AD disturbances, including mitochondrial dysfunction, neuroinflammatory response, oxidative stress, neurotoxic substance deposition, and memory deficits. Here, we will introduce them to AD one by one in the following.

### N6-Methyladenosine and Mitochondrial Dysfunction of Alzheimer’s Disease

The central nervous system requires approximately 20% of the body’s total basal oxygen consumption to support neuronal energy expenditure. Mitochondria are organelles that are responsible for energy production. Therefore, neurons are damaged by mitochondrial deficiency. Mitochondrial dysfunction was observed in the brains of AD patients, even before the appearance of neurofibrillary tangles and senile plaques (Lim et al., 2020).

Some m^6^A-related enzymes have been shown to affect mitochondrial function. The physiological role of METTL3 in mitochondria is under debate. Shi et al. (2021) considered that METTL3 preserved mitochondrial function in Down syndrome by reducing the expression of nuclear receptor-interacting protein 1 (NRIP1), a crucial gene in the regulation of the mitochondrial pathway. But Zhang et al. (2021) demonstrated that METTL3 and YTHDF2 cooperatively promoted mitochondrial dysfunction and inflammatory response during oxLDL-induced inflammation in monocytes. In addition, the demethylase FTO inhibitor MO-I-500 was found to ameliorate astrocyte mitochondrial dysfunction in streptozotocin-induced AD cell models (Cockova et al., 2021).

### N6-Methyladenosine and Neuroinflammatory Response of Alzheimer’s Disease

An excessive neuroinflammatory response is harmful for the brain. Growing evidence shows that neuroinflammation is directly implicated in AD processes (Amor et al., 2014). Microglia are the main effectors in the neuroinflammatory process. Once overactivated, microglia may release proinflammatory cytokines and accelerate neurodegeneration.

Li Q. et al. (2021) identified a distinct m^6^A epitranscriptome in microglia. They found that m^6^A served as a novel and essential regulator of the anti-inflammatory and proinflammatory responses of microglia. An *in silico* analysis of immunoprecipitated methylated RNAs with microarrays also demonstrated that m^6^A methylation was increased in major inflammatory pathways (Chokkalla et al., 2019), indicating that RNA m^6^A methylation is closely related to neuroinflammation. Indeed, METTL3 was found to promote lipopolysaccharide (LPS)-induced neuroinflammation through the TRAF6/NF-κB pathway (Wen et al., 2020). LPS-induced neuroinflammation was considered might impair the efficient readout of neuronal genetic information and might contribute to a progressive disruption in the readout of genetic information in the AD brain (Zhao et al., 2017). The METTL3 knockdown was found to inhibit the inflammatory response by regulating the variable splicing of MyD88 (Feng et al., 2018).

### N6-Methyladenosine and Oxidative Stress of Alzheimer’s Disease

Oxidative stress plays a crucial role in AD pathogenesis. The brain is more vulnerable to oxidative stress than other organs, and most of the components of neurons (proteins, lipids, and nucleic acids) can be oxidized in AD (Bonda et al., 2010).

It has been reported that m^6^A modification is affected by oxidative stress. The arsenic exposure hypothesis for AD provides a parsimonious testable hypothesis for the development and progression of this devastating disease to some degree (Gong and O’Bryant, 2010). Arsenite-induced oxidative stress possibly increases the levels of RNA m^6^A methylation by regulating m^6^A “Writers” or “Erasers,” particularly promoting METTL14 and WTAP expression (Zhao et al., 2019). Additionally, m^6^A modification is important in the regulation of oxidative stress. The m^6^A-binding protein YTHDF1/3 was found to promote stress granule formation (Fu and Zhuang, 2020). Although associations *per se* cannot prove cause-effect relationships, the development of pathological stress granules has been implicated in the onset and progression of AD (Ash et al., 2014). The m^6^A methyltransferase METTL3 was reported to attenuate oxidative stress and cell apoptosis in colistin-induced kidney injury by activating the antioxidant Keap1/Nrf2 pathway (Wang et al., 2019). The reduced neuronal m^6^A modification in the hippocampus caused by METTL3 knockdown led to extensive synaptic loss and neuronal death along with multiple AD-related cellular alterations, including oxidative stress and aberrant cell cycle events *in vivo* (Zhao et al., 2021).

### N6-Methyladenosine and Pathologic Hallmark of Alzheimer’s Disease

Accumulation of insoluble neurotoxic aggregates, including extracellular amyloid (A)β plaques and intracellular tau neurofibrillary tangles, represents a major pathological hallmark of AD. Their accumulation leads to neuronal degeneration, synaptic dysfunction, and ultimately, dementia (Lane et al., 2018).

#### N6-Methyladenosine and Aβ Plaques

Folic acid, a water-soluble B vitamin, reduces the production of Aβ and slows the progression of AD by involving in generating S-adenosylmethionine (SAM), potentially enhancing the levels of RNA m^6^A methylation (Li et al., 2015; Li N. et al., 2021), implying that RNA m^6^A methylation may be involved in Aβ metabolism. Interestingly, Aβ treatment was found to have significantly reduced METTL3 and postsynaptic density-95 (PSD-95) expression in rat primary cortical neurons. On the contrary, METTL3 overexpression was found to rescue Aβ-induced synaptic PSD-95 loss *in vitro*. Importantly, METTL3 overexpression rescued synaptic damage and cognitive impairment in Aβ-induced AD mice. In addition, the demethylase FTO was reported to alleviate Aβ-induced cell degeneration via the PKA/CREB signaling pathway (Hu et al., 2020). In addition to targeting Aβ, m^6^A methylation also altered the expression levels of Aβ production-related proteins, such as Aβ precursor protein (APP) and the β-site APP-cleaving enzyme (BACE1) (Kolisnyk et al., 2017; Edens et al., 2019). The m^6^A “Reader” FMRP was found to regulate the local protein synthesis of neuronal synapses and change the nuclear output of m^6^A-dependent mRNA by regulating APP mRNA translation (Chang et al., 2017; Edens et al., 2019). The downregulation of the “Reader” HNRNPA2B1 was shown to promote abnormal splicing of BACE1 (Kolisnyk et al., 2017).

#### N6-Methyladenosine and Tau Neurofibrillary Tangles

In human postmortem AD samples, Huang et al. (2020) observed METTL3 accumulation in the insoluble fractions, which correlated positively with levels of insoluble tau neurofibrillary tangles. This was accompanied by an increased level and redistribution of METTL3 expression in the AD hippocampus, likely representing aberrant misfolding and/or aggregation of METTL3, perhaps similar to the frequent aggregation of misfolded proteins in AD (Huang et al., 2020). In the brain of an AD mouse model, upregulated FTO was also observed to activate the phosphorylation of tau and accelerate the pathological hallmarks of AD in an mTOR-dependent manner (Li et al., 2018). In the *Drosophila* AD model, which specifically expresses the human tau gene with the R406W mutation in the eye, found that the loss of m^6^A by loss of METTL3, METTL14 or YTHDF enhanced tau toxicity and had more severe locomotive defects (Shafik et al., 2021).

### N6-Methyladenosine and Memory Disorder in Alzheimer’s Disease

Learning and memory impairments are the most important clinical symptoms of AD. Synaptic plasticity, that is, the adjustability of synaptic morphology and strength, is considered to be the basis of learning and memory. It is worth noting that short- and long-term memory requires multiple layers of regulation, from protein modifications at the synapse to RNA synthesis *de novo* in the nucleus (Cai et al., 2016; Holtmaat and Caroni, 2016; Karunakaran et al., 2016). The stability and translation of RNA transcripts have been shown to depend on m^6^A modification (Yue et al., 2015), which is strongly biased to neuronal genes and functions (Meyer et al., 2012; Schwartz et al., 2014). Thus, it is perhaps unsurprising that RNA m^6^A methylation modifications have been implicated in neural plasticity, thereby affecting learning and memory.

#### N6-Methyladenosine Methyltransferase and Memory Disorders

Increased m^6^A levels in adult neurons have been found to promote the transcriptome response to synaptic plasticity (Engel et al., 2018; Leighton et al., 2018). In contrast, reducing the m^6^A peaks in cellular mRNAs by knocking out some m^6^A methyltransferases, such as METTL3, METTL14, METTL16, WTAP, RBM15/15B, and HAKAI may result in memory disorders (Ping et al., 2014; Schwartz et al., 2014; Růžička et al., 2017; Knuckles et al., 2018; Zhang et al., 2018; Shafik et al., 2021). The most studied m^6^A “Writer,” METTL3, has been shown to have a direct effect on the regulation of hippocampal-dependent memory formation. The overexpression of METTL3 in the dorsal hippocampus of wildtype mice was found to enhance long-term memory consolidation significantly (Zhang et al., 2018; Table 1), whereas the knockout of METTL3 in the forebrain was found to inhibit memory consolidation, which could be restored by adequate training (Zhang et al., 2018; Zhao et al., 2021). METTL14-mediated RNA m^6^A modification is also critical for epitranscriptomic regulation of learning. The deletion of METTL14 was observed to reduce striatal m^6^A levels, increase neuronal excitability, and severely impair striatal-mediated learning-related behavior (Table 1; Koranda et al., 2018).

**TABLE 1:** The function of RNA N6-methyladenosine methylation modification enzyme in learning and memory.

Types	Name	Roles	References
Methyltransferase “Writers”	METTL3	Enhance the long-term memory consolidation by promoting m^6^A methylation.	Zhang et al., 2018; Huang et al., 2020
	METTL14	Mediated RNA m^6^A modification is critical for striatum function and epitranscriptomic regulation of learning.	Koranda et al., 2018
Demethylase “Erasers”	FTO	Limit memory formation by inhibiting m^6^A methylation; A gene variant of FTO has been found to be a risk factor in AD.	Ho et al., 2010; Keller et al., 2011; McTaggart et al., 2011; Reitz et al., 2012; Li L. et al., 2017; Walters et al., 2017; Engel et al., 2018
Methylation binding protein “Readers”	YTHDF1	Enhance memory formation by promoting translation process of target transcripts in a way of neuronal stimulating dependence.	Shi et al., 2018
	YTHDF3	Increase dendritic spine density and promote synaptic transmission.	Merkurjev et al., 2018
	FMRP	Regulates the translation of some functional synaptic proteins	Huber et al., 2002
	HNRNPA2B1	The selective loss in entorhinal cortex leads to aberrant alternative splicing and dendritic loss.	Berson et al., 2012
	PRRC2A	Control the specification and myelination of oligodendrocyte and improve cognitive deficits.	Wu et al., 2019

#### N6-Methyladenosine Demethylase and Memory Disorders

Precise RNA m^6^A modification is necessary for memory formation, thereby implicating m^6^A demethylase concurs with memory regulation. Despite being ubiquitously expressed, FTO, the best characterized “Eraser,” is enriched in the nuclei and dendrites, and near dendritic spines of mouse dorsal hippocampal CA1 neurons (McTaggart et al., 2011). The expression of FTO protein decreased shortly after a situational fear reflex, which implies that FTO typically limits memory formation (Table 1; Walters et al., 2017). Indeed, knocking out FTO in the prefrontal cortex of mice was found to enhance fear memory consolidation, with m^6^A modification on several fear-related genes significantly increased (Li L. et al., 2017; Engel et al., 2018). However, a gene variant of FTO was found to be a possible risk factor for AD (Table 1; Ho et al., 2010; Keller et al., 2011; Reitz et al., 2012). A prospective cohort study by Keller et al. (2011) suggested that the FTO AA-genotype had a higher risk for AD compared to TT-carriers. Reitz et al. (2012) used 1,877 Caucasian cases and controls from the NIA-LOAD study and 1,093 Caribbean Hispanics to further explore the association of FTO with AD. They found that genetic variation in Introns 1 and 2 of the FTO gene may contribute to AD risk (Reitz et al., 2012). The aforementioned studies suggested that maintaining a low and basic expression level of FTO in AD might be necessary for precise RNA m^6^A modification.

#### RNA N6-Methyladenosine Methylation-Binding Protein and Memory Disorders

N6-methyladenosine methylation-binding proteins also make marked contributions to memory storage. Interrupting m^6^A-mediated function via knockdown of m^6^A “Readers” in hippocampal neurons resulted in synaptic dysfunction, including immature spine morphology, and destroyed excitatory synaptic transmission, accompanied by decreased clusters of PSD-95 and reduced surface expression of the AMPA receptor subunit GluA1 (Merkurjev et al., 2018).

YTHDF1 is mainly expressed in the hippocampus and promotes the translation of target transcripts through neuronal stimulation. YTHDF1-knockout mice showed impaired learning and memory, reduced synaptic transmission, and decreased long-term potentiation (Table 1; Shi et al., 2018). Interestingly, these phenotypes were similar to those obtained with METTL3 depletion (Shi et al., 2018), suggesting “Readers” and “Writers” can, to some extent, phenocopy each other in the brain. In YTHDF3-knockdown neurons, excessive dendritic filopodia in place of mature spines were observed, and a decreased percentage of spines containing a PSD-95 cluster and surface GluA1 expression was observed (Table 1; Merkurjev et al., 2018). In addition, FMRP was found to regulate the translation of some functional synaptic proteins (Table 1; Huber et al., 2002). The absence of FMRP in Fragile-X Syndrome causes excessive and persistent protein synthesis in dendrites, leading to an excess number of dendritic spines and synaptic dysfunction (Bassell and Warren, 2008; Richter et al., 2015). HNRNP was found to be relatively highly expressed in brains with a high metabolism. The selective loss of HNRNPA2B1 in the entorhinal cortex led to aberrant alternative splicing and dendritic loss (Table 1; Berson et al., 2012). PRRC2A has been shown to control the specifications and myelination of oligodendrocytes. PRRC2A-knockout mice showed cognitive deficits (Table 1; Wu et al., 2019).

## Conclusion and Outlook

RNA m^6^A methylation, which is abundant in the mammalian brain, is a significant epitranscriptomic modification. m^6^A has a wide range of effects on AD and can be precisely regulated. The emergence of cross-talk between m^6^A “Writers,” “Erasers,” and “Readers” makes it more complicated. The most appropriate example is METTL3. METTL3 is a key component of the m^6^A methyltransferase complex, with its eminent methyltransferase activity, but has also been reported to function as an m^6^A “Reader.” Studies have reported that METTL3 directly promotes the translation of several m^6^A-modified mRNAs, such as the Hippo pathway effector TAZ and the epidermal growth factor receptor, by interacting with translation initiation machinery, independent of its methyltransferase and downstream m^6^A “Reader” activity (Lin et al., 2016). Hence, METTL3 might be both an m^6^A “Writer” that methylates mRNA along with other members of the methyltransferase complex, by identifying unmethylated mRNA, and an m^6^A “Reader” that enhances mRNA translation alone, by identifying methylated mRNA. The second pertinent example is METTL16, which interacts with MAT2A hairpins to regulate MAT2A through two mechanisms: reducing mRNA stability in SAM-sufficient conditions and promoting pre-mRNA splicing in SAM-limiting conditions. The former relies on METTL16 recognition of methylated MAT2A pre-mRNA. As the sites of m^6^A on MAT2A pre-mRNA are occupied, METTL16 is quickly separated from MAT2A pre-mRNA, increases retention of the last intron in MAT2A pre-mRNA, and reduces its stability. However, in SAM-limiting conditions, METTL16 binds to unmethylated MAT2A pre-mRNA to promote the splicing of MAT2A pre-mRNA by recruiting the cleavage factor I_*m*_ complex (CFIm), and finally increases the expression of MAT2A mature mRNA (Scarborough et al., 2021). In brief, the effects of METTL3 and METTL16 likely reflect the typical “Writer—Reader” paradigm. More importantly, their effects are precisely regulated. The activity of METTL3 is controlled by post-translational modifications, such as SUMOylation (Liu et al., 2020), and METTL16 is regulated by intracellular SAM levels, which makes the regulatory network extremely intricate.

In addition, differences in experimental conditions and animal models increase discrepancies in research results. Some studies have shown a reduction in m^6^A modification in AD (Shafik et al., 2021) and Parkinson’s disease models (Chen et al., 2019). However, Han et al. (2020) found that m^6^A methylation was elevated in the cortex and hippocampus of an AD model. The variation tendency of METTL3 and FTO in AD brain were also contradictory between the studies by Han et al. (2020), Shafik et al. (2021). Shafik et al. (2021) found METTL3 was downregulated and FTO was upregulated, which was in contrast to the study by Han et al. (2020) These discrepancies might be due to differences in the animal models employed. 9-month-old APP/PS1 mice and 6-month-old mice were used by Han et al. (2020), Shafik et al. (2021), respectively. Given that Shafik et al. also observed significantly more m6A sites as age increases during the aging process in both mouse and human brain areas, we speculate the increase in m6A methylation in that the study of Han et al. (2020) may be more likely to be compensatory and early changes in AD. But, of course, more comprehensive experiments are required to elucidate the changes of RNA m^6^A methylation modification in the various stages of AD.

## Author Contributions

RZ contributed to the design of the review and drafted the manuscript. YZ and FG contributed to revision of the manuscript. SL and HC contributed to the design of the review and critical revision of the manuscript and had primary responsibility for the final content. All authors contributed to the article and approved the submitted version.

## Conflict of Interest

The authors declare that the research was conducted in the absence of any commercial or financial relationships that could be construed as a potential conflict of interest.

## Publisher’s Note

All claims expressed in this article are solely those of the authors and do not necessarily represent those of their affiliated organizations, or those of the publisher, the editors and the reviewers. Any product that may be evaluated in this article, or claim that may be made by its manufacturer, is not guaranteed or endorsed by the publisher.
